# Supplementary material for: SPRING, an effective and reliable framework for image reconstruction in single-particle Coherent Diffraction Imaging
Source: NPJ Comput Mater. 2025 Aug 19;11(1):265. doi: 10.1038/s41524-025-01661-y (PMC12364707; doi:10.1038/s41524-025-01661-y)
Supplement: Supplementary file 1 — Supplemental information [file 41524_2025_1661_MOESM1_ESM.pdf]

SPRING, an effective and reliable framework for image reconstruction in single-particle Coherent Diffraction Imaging

Supplementary Material

1 Further details on experimental data and data selection

Samples are injected in vacuum, and are intercepted by the FEL pulse in its focus, of size of few $\mu\mathrm{m}$. The presence of the sample in the focus is based on statistics. The probability for the pulse to hit a sample is defined as *hitrate*, which is the ratio between the number of shots with diffraction signal (*hits*) and the total number of acquired shots. As it is necessary to statistically ensure that most *hits* are produced by a single particle, the particle density in the experimental chamber is controlled to keep the *hitrate* typically below $5\,\%$ (i.e., most of the acquired raw data is corresponds to *dark* images with no sample in the interaction region).

Hits can be easily identified by selecting those images whose total signal on the detector is above a given threshold. Still, the overall brightness of the hits (proportional to the total number of photons scattered on the detector) has large variations, usually up to few orders of magnitude. These intensity fluctuations are mainly caused by shot-by-shot jitter in the FEL pulse energy and by the shape of the FEL focus.

The diffraction data treated in this work have been selected among the brightest *hits*, which represents only a fraction of the total detected hits in a dataset. The brightest hits are the most valuable, as they provide the information on the samples at the highest spatial resolution available and are easier to treat for phase retrieval algorithms.

As low-brightness, sub-optimal diffraction images are the largest fraction of the detected *hits*, the analysis of those patterns can be anyway relevant to increase the information extracted from a CDI experiment, especially in the case of investigations based on statistical observations. A brief discussion on SPRING performance on this type of data is reported in Sec. 3.1.

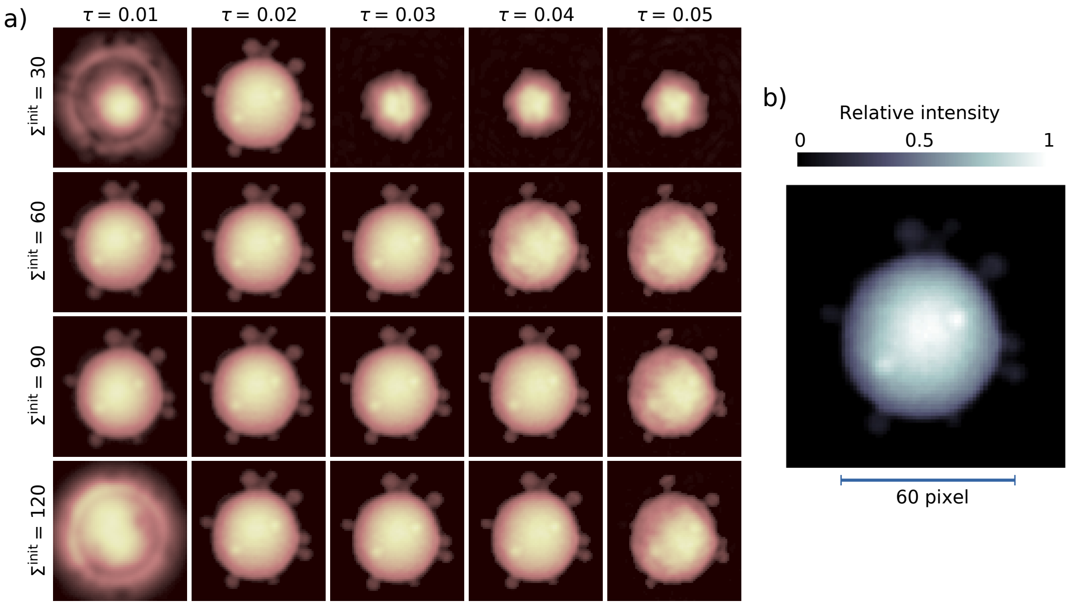

Supplementary Figure 1: Imaging results for different combinations of the initial support size Σ_{init} and threshold value τ, shown in a), highlighting the wide range of settings for which MPR still correctly identifies the solution. In b), the solution is represented in a perceptually linear color scale, to highlight the different brightness of the nanocluster components. The experimental diffraction pattern is taken from Dataset A (see Fig. 2).

2 Stability over algorithms' parameters

This section investigates the stability and resiliency of the MPR approach against different values of the parameters of iterative algorithms and of the Swrink-wrap (SW) method to retrieve the support function.

2.1 Retrieval of the support function

The first test demonstrates how the success of a reconstruction depends on the starting support size Σ_{init}, indicated as a dimension in pixels (see Appendix IV G), and the threshold for the support update τ [1]. Other parameters are kept to their default value indicated in Table I. Supplementary Figure 1 shows the result of this benchmark, where different combinations of Σ^{init} and τ are tested. In particular, the starting support sizes, 30, 60, 90 and 120 span from around the size of the sample, indicated in Supplementary Figure 1b as 60 pixel, to up to twice that value. This is tested against different threshold values for the support update, from as little as $\tau = 0.01$ up to $\tau = 0.05$. At the smallest support size, the initial span of the support is much smaller than the correct one, implying that the algorithm has to enlarge the support area instead of shrinking, which is not what the SW algorithm is designed for. Still, the reconstruction

process manages to converge to the solution, even if only in the case of an "optimal" threshold value $\tau = 0.02$.

For what concerns the higher values of τ, reconstructions fail at $\tau = 0.05$. However, such a behavior can derive from intrinsic features of the sample. The sample has, in fact, a hailstone-like shape, with small sub-clusters surrounding one which is 10 to 20 times larger. The relative brightness of the small components in the 2D projection is thus only few percent of the main sub-cluster, close to the threshold value $\tau = 0.05$. This large dynamic range required for the reconstruction can be appreciated better in Supplementary Figure 1b, where the reconstruction is represented in a perceptually linear color scale. For what concerns the lowest values of $\tau = 0.01$, the convergence of the algorithm is sensitive to the starting support size, and fails for 30 pixel (half the size of the real sample) and 120 (twice the size).

It is worth noting two important aspects of the results in Supplementary Figure 1. First, there is an "optimal" threshold value $\tau = 0.02$ for which the reconstruction converged for all support sizes. This flexibility allows for reconstructing samples of greatly varying size taken at similar conditions. Second, the capability of retrieving the correct sample shape with low threshold values (even as low as 0.01 when the initial support size is not far from the real sample size) allows the imaging of samples with a particularly high contrast between the different components.

2.2 Parameters of the iterative algorithms

Other settings that usually need a certain amount of case-by-case optimization are the ones involving iterative algorithms (IAs). Here we report how the MPR approach is robust against variations and sub-optimal choices of those settings.

All IAs require a certain starting number of iterations it, which is typically reduced to 0 at the end of the reconstruction process to improve the stability of the reconstructions [2]. Two IAs are currently implemented in MPR, the Hybrid Input-Output algorithm (HIO) [3] and the Relaxed-Averaged Alternating Reflections (RAAR) [4], both depending on a so-called *feedback parameter* β [3, 4]. Different values of β correspond to different behavior of the algorithms (i.e. different trajectories in the parameters' space [2]), which often have to be tuned depending on the type of data.

Supplementary Figure 2 shows reconstruction results for varying number of iterations it and feedback parameter β. Supplementary Figure 2a reports the test for the HIO algorithm, with data from Dataset A. Supplementary Figure 2b reports, instead, the imaging result obtained via the RAAR algorithm, on data from dataset B. For both tests, the threshold value τ for the support update was set to 0.03 and the initial support size was set to 90. Other parameters are set to the default values indicated in Table I. In both cases, the correct identification of the sample's shape appears to be independent of the feedback parameter β. The same observation applies to the starting number of iterations it, for which a successful reconstruction is achieved for either HIO or RAAR iterations with as little as $it = 5$. This result highlights well the action of the crossover operation. In fact, the use of IAs like HIO and RAAR is necessary to better explore

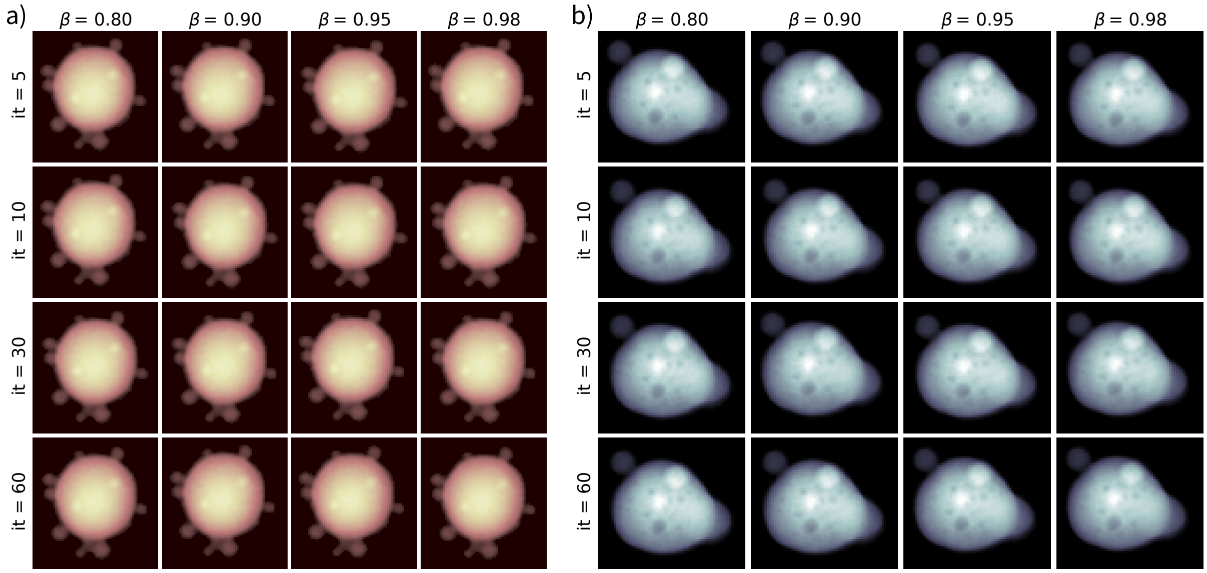

Supplementary Figure 2: Imaging results for different number of IA iterations and value of the parameter β, showing the resiliency of the MPR approach against sub-optimal settings. In a), the HIO algorithm is tested on Dataset A. In b), the RAAR algorithm is tested on dataset B.

the parameter space, as discussed in Sec. II A. Within MPR, part of this task is undertaken by the crossover operator (see Sec. II B and Appendix IV C), allowing for the identification of the solution even with a reduced number of IA iterations, executed in the *Self-improvement* step (see Fig. 1).

3 Further comparisons with conventional methods

In this section we further compare the behavior of SPRING with the results obtained by the conventional approach. As discussed in Sec. II E of the main manuscript, the conventional use of phase retrieval algorithms is to run a number of independent reconstructions and then keep those results that reached the lowest error value. This approach can be achieved with SPRING by switching off the *Crossover* and *Selection* operations in the main loop (compare Alg. 1 and Alg. 3 in the main manuscript). In this way, the population of initial reconstructions $\{\mathcal{R}^p\}$ are independently carried on for all generations G without sharing information with the *genetic operations*. Here we refer to the conventional use of iterative algorithms as IPR.

As discussed in the main manuscript, for a reconstruction via MPR with population size P, the corresponding conventional IPR is performed with population size $2P$. This is done to make the comparison fair in terms of execution time and computational costs, as the MPR method executes local optimization with iterative algorithms on two populations, $\{\mathcal{R}^p\}$ and the one created by the crossover operation $\{\mathcal{R}^p_{\text{new}}\}$. For these tests, the MPR population has been

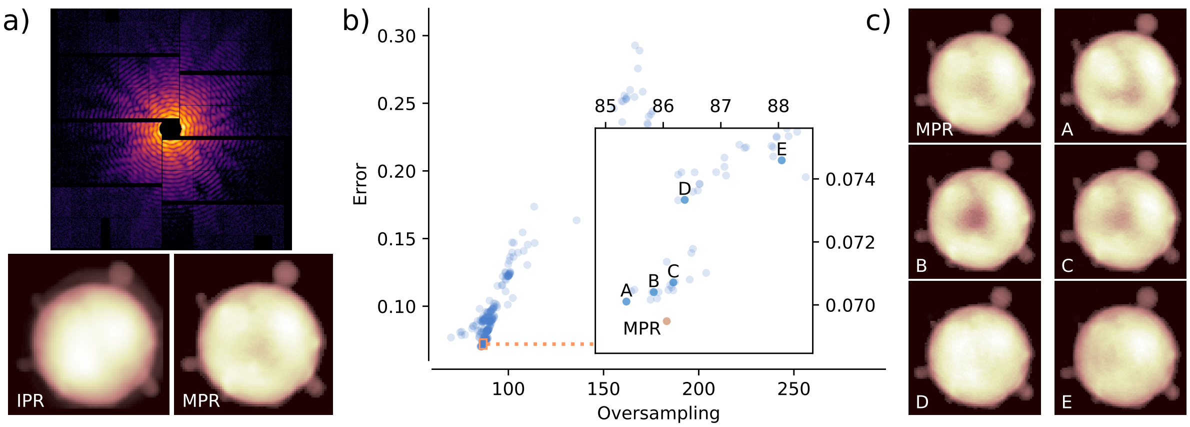

Supplementary Figure 3: Reconstruction example from dataset A performed with the conventional IPR approach and the SPRING implementation of MPR. Sub-figure a) shows the experimental diffraction data along with the average reconstructions, i.e. average density calculated over the whole population independently on the final error value. The average reconstruction is not a good indicator for the IPR performance, as typically only a fraction of the reconstructions reach the correct convergence. For this reason, a more detailed inspection on the IPR performance is reported in b) and c). Sub-figure b) gives an overview on the distribution of the individual reconstructions $\mathcal{R}^p$ depending on the final error value (y-axis) and support size (x-axis). The support size is reported in terms of the oversampling degree, calculated as the ration between the number of pixels in the matrix and the number of pixels where the support function $\mathcal{R} \triangleright \mathrm{s}_{ij} = 1$ (i.e. the area of the support, see Eq. (5) in the main manuscript). The inset plot in b) is a zoom in the region where the reconstructions reached proper convergence, where also the best reconstruction in the MPR population is reported as a red dot. The SPRING result with MPR, along with 5 different reconstructions of IPR selected around the convergence region are marked in the plot and their densities $\mathcal{R} \triangleright \rho$ are shown in c).

set to $P = 128$ (i.e. $P = 256$ for the conventional IPR).

Supplementary Figures 3, 4, 5 and 6 report a comparison test performed on different diffraction patterns belonging to the Dataset A and B described in Sec. II C of the main manuscript.

In Supplementary Figure 3, the conventional IPR method reveals acceptable performance, i.e. many of the reconstructions reached a low error value at the correct oversampling value, indicating a correct identification of the sample shape. This is visible not only from the plot in Supplementary Figure 3b, but also from the individual reconstructions reported in Supplementary Figure 3c. The large density fluctuations visible in the different densities of Supplementary Figure 3c are due to the presence of *loosely constrained modes*, in particular the *gaussian* mode, arising due to the lack of diffraction data in the center of the pattern (discussed in Sec. IV O and thoroughly investigated in Ref. [5]).

The IPR method struggles significantly more for the example shown in 4. Only few IPR

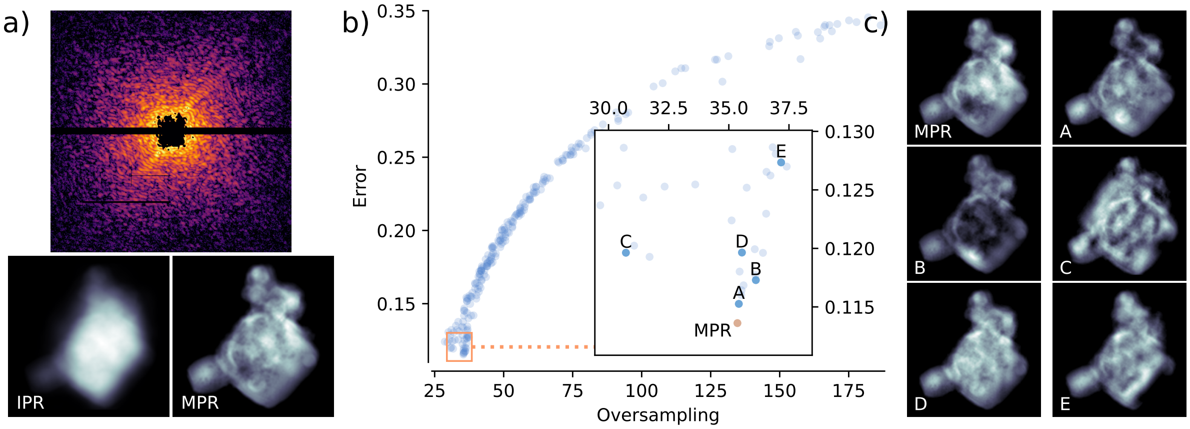

Supplementary Figure 4: Reconstruction example from dataset B performed with the conventional IPR approach and the SPRING implementation of MPR. The figure is organized as Supplementary Figure 3.

reconstructions are reaching proper convergence, and among those the density fluctuations are evident, hiding in some cases main features of the sample under study.

Supplementary Figure 5 reports the comparison for diffraction data discussed in Sec. II G of the main manuscript, and it differs from the other examples for the significant phase shift expected in the density due to the photon energy being resonant with xenon. In this case, not even a single reconstruction reached proper convergence with conventional IPR. It is worth noting that, in all the three cases shown here, the error of the best SPRING reconstruction with the MPR method is systematically lower than the best of the conventional IPR.

The final test in Supplementary Figure 6 is instead concerning a diffraction data that is easy to reconstruct for the conventional IPR. This is mainly due to high brightness of the diffraction and the small size of the sample (i.e. high oversampling). In this case, also the average IPR reconstruction shown in Supplementary Figure 6a displays a meaningful density distribution, because most of the reconstruction attempts in the population reached proper convergence. This is well visible also in Supplementary Figure 6b and Supplementary Figure 6c. Despite the ideal conditions for the conventional IPR, the error of the best MPR reconstruction again reaches a lower error value, indicating that that the optimization task is better performed by SPRING when the neighborhood of the solution is reached.

3.1 Comparison for noisy data

As mentioned in the main text, there is little control over the brightness of the diffraction image in a typical CDI experiment. The main reasons for this are the intensity distribution in the FEL focus, commonly approximated with a Gaussian profile, and the intrinsic shot-by-shot jitter of the total pulse energy. Diffraction images with low signal pose a problem for reconstruction

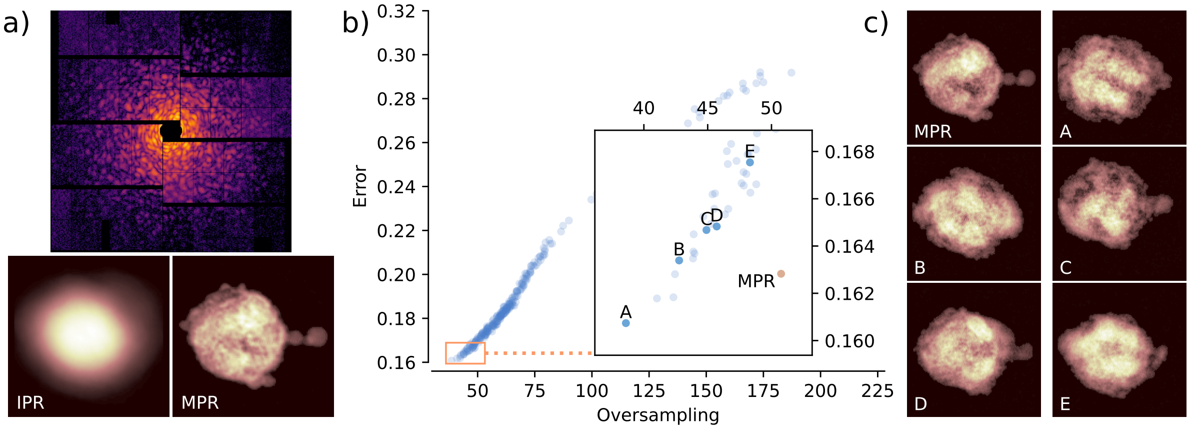

Supplementary Figure 5: Reconstruction example from dataset A performed with the conventional IPR approach and the SPRING implementation of MPR. The figure is organized as Supplementary Figure 3.

algorithms.

The actual spatial resolution of a reconstruction depends on the maximum momentum transfer at which usable signal is recorded on the detector. Thus, patterns with less photons intrinsically imply a lower effective resolution in the reconstruction. It is then of great importance to maximize the information extracted at high momentum transfer. An example of imaging from noisy data is reported in Supplementary Figure 7. In this example, the sample has a relatively small size, which allows for the convergence of most of the independent IPR reconstructions to the correct sample shape. Due to the low Signal-to-Noise Ratio (SNR) in the data, the individual reconstructions reported in Supplementary Figure 7c are affected by relatively high noise, which makes it hard to distinguish inner, high-resolution, features of the particle. However, the actual features of the sample arise well when the average reconstruction is calculated, as shown in Supplementary Figure 7a. The better convergence performance of MPR is reflected there in a higher level of detail, as well as in an increased contrast of the high resolution features.

A further consequence of low-brightness patterns concerns the reduced *field of view* of the CDI method. As discussed in the main text, the missing data at low transfer momentum (close to the center of the pattern) limits the maximum size of the samples that can be reliably retrieved. This is because algorithms have to "guess" the missing data from the known one, which leads to instabilities as the sample gets larger (see Sec. II F of the main text).

As the missing diffraction data has to be retrieved from the known recorded intensities, the lower SNR renders this a much harder tasks for iterative algorithms and amplifies their instabilities. An example of this is reported in Supplementary Figure 2, where conventional IPR struggles in finding the correct sample morphology even for those reconstructions that reached an oversampling value close to the correct one.

The higher capability of MPR in dealing with sub-optimal diffraction patterns allows to treat

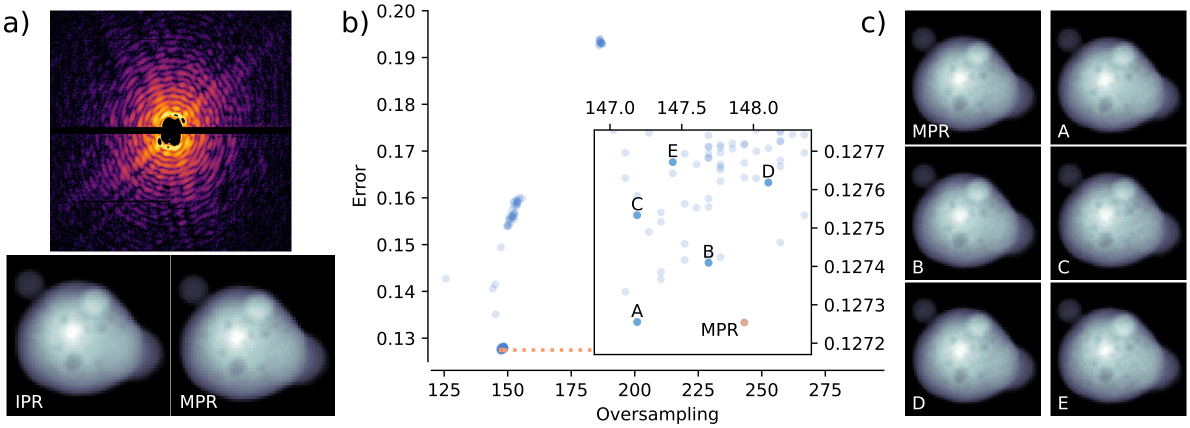

Supplementary Figure 6: Reconstruction example from dataset B performed with the conventional IPR approach and the SPRING implementation of MPR. The figure is organized as Supplementary Figure 3.

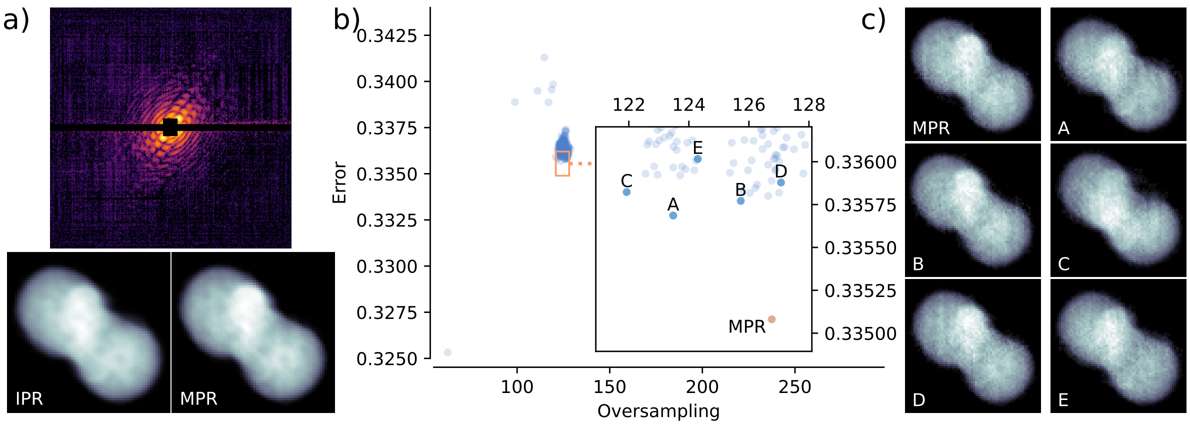

Supplementary Figure 7: Reconstruction example from dataset B performed with the conventional IPR approach and the SPRING implementation of MPR. The figure is organized as Supplementary Figure 3. In this example, the diffraction data, reported in a), is characterized by a low number of photons which limits the maximum momentum transfer at which useful information is acquired. Furthermore, the diffraction pattern is affected by *straylights*, visible as a bright straight signal on the right side close to the gap between the two detector module. Due to the low overall signal strength, also background noise coming from the detector's electronics is visible in the darker regions.

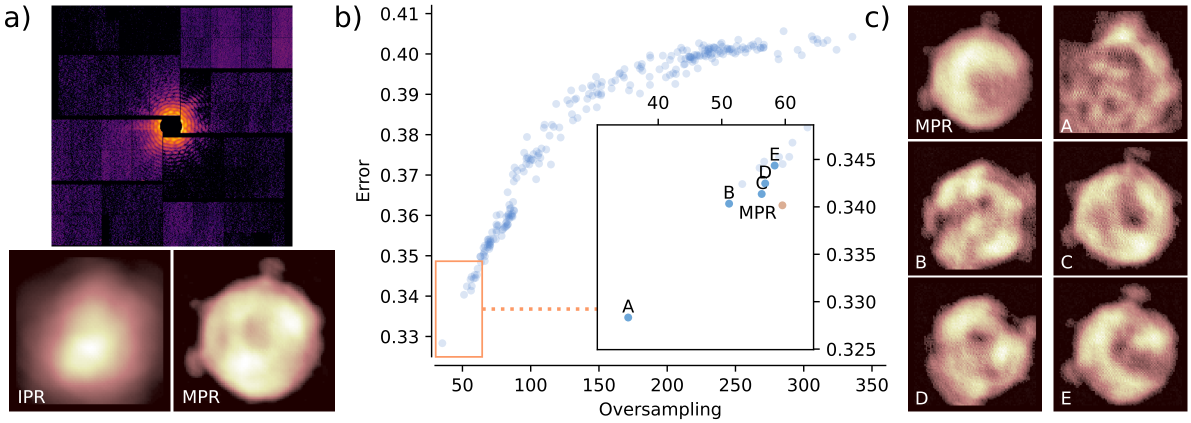

Supplementary Figure 8: Reconstruction example from dataset B performed with the conventional IPR approach and the SPRING implementation of MPR. The figure is organized as Supplementary Figure 3. In this example, the diffraction data, reported in a), has a particularly low signal and it is produced by a large sample.

a larger fraction of the acquired data, practically increasing the experiment output. Furthermore, this higher stability allows for the design of single-particle single-shot CDI experiments with samples of lower scattering cross-section, as well as for the use of higher photon energies, where the scattering cross-section of materials is significantly weaker.

Supplementary References

[1] S. Marchesini, H. He, H. N. Chapman, S. P. Hau-Riege, A. Noy, M. R. Howells, U. Weierstall, and J. C. Spence, "X-ray image reconstruction from a diffraction pattern alone," *Physical Review B*, vol. 68, no. 14, p. 140101, 2003.

[2] S. Marchesini, "Invited article: A unified evaluation of iterative projection algorithms for phase retrieval," *Review of scientific instruments*, vol. 78, no. 1, p. 011301, 2007.

[3] J. R. Fienup, "Phase retrieval algorithms: a comparison," *Applied optics*, vol. 21, no. 15, pp. 2758–2769, 1982.

[4] D. R. Luke, "Relaxed averaged alternating reflections for diffraction imaging," *Inverse problems*, vol. 21, no. 1, p. 37, 2004.

[5] P. Thibault, V. Elser, C. Jacobsen, D. Shapiro, and D. Sayre, "Reconstruction of a yeast cell from x-ray diffraction data," *Acta Crystallographica Section A: Foundations of Crystallography*, vol. 62, no. 4, pp. 248–261, 2006.
